# Sensitization patterns and minimum screening panels for aeroallergens in self-reported allergic rhinitis in China

**DOI:** 10.1038/s41598-017-10111-9

**Published:** 2017-08-24

**Authors:** Hongfei Lou, Siyuan Ma, Yan Zhao, Feifei Cao, Fei He, Zhongyan Liu, Jean Bousquet, Chengshuo Wang, Luo Zhang, Claus Bachert

**Affiliations:** 1 Department of Otolaryngology Head and Neck Surgery, Beijing TongRen Hospital, Capital Medical University, Beijing, 100730 China; 2 Beijing Key Laboratory of nasal diseases, Beijing Institute of Otolaryngology, Beijing, 100005 China; 3 Department of Allergy, Beijing TongRen Hospital, Capital Medical University, Beijing, 100730 China; 4 Department of Respiratory Diseases, University Hospital, Hôpital Arnaud de Villeneuve, Montpellier, France; 5 Upper Airways Research Laboratory, Department of Oto-Rhino-Laryngology, Ghent University Hospital, De Pintelaan 185, 9000 Ghent, Belgium

## Abstract

Optimization of skin prick test (SPT) panels, especially in view of significant differences in sensitizations patterns within different geographical areas, is an unmet need within China. Our aim was to assess the patterns and clinical relevance of aeroallergen sensitizations in allergic rhinitis (AR) and define the minimal battery of SPT allergens, classified according to the Köppen-Geiger climate map. Overall, 7148 subjects with self-reported AR completed a standard questionnaire and were assessed for sensitization to relevant allergens by SPT. 6340 (88.7%) patients had at least one positive skin prick reaction, and demonstrated unique sensitization patterns by stratification with age, gender, and geographic region. Sensitization to house dust mites (HDM) was highest in south China, whereas the three most prevalent aeroallergens were mugwort, ragweed and dandelion pollen in north-west China. Higher sensitization rates and multiple sensitizations were associated with AR comorbidities. Eight allergens (*Der f*, mugwort, *Blatella*, hazel, goosefoot, *Penicillium notatum*, animal dander and *Der p*) allowed identification >96% of sensitized subjects in central China. Differences in optimal panels were observed between regions, with five to six allergens being sufficient for north-east, north-west and south China. These SPT panels may provide a cost-effective tool for screening sensitized patients in China.

## Introduction

Allergic rhinitis (AR) is an increasing challenge to public health worldwide^1^. Self-reported AR prevalence in China has increased from 11.1% to 17.6% over a 6-year period^2, 3^. AR negatively impacts quality of life and imposes a considerable burden on society. The detection of the causative aeroallergen is thus important for an accurate diagnosis and appropriate management of AR.

Aeroallergen sensitization varies widely between geographical areas, depending on exposure^3–5^. Previously, mathematical spatial models were used to estimate the epidemic patterns^6–9^. Both long distance travel and increased transportation rates associated with modern day living as well as migration may exert prominent effects on the formation of sensitization patterns within the population^4, 9–11^. Additionally, climate changes could affect sensitization profiles by altering aeroallergen distribution, amount, germination rate, and allergenicity^4, 10^. Similarly, gender^12, 13^ and age^14–16^ may modify sensitization patterns. Furthermore, polysensitization has been shown to strongly increase the presence of AR symptoms and predict the subsequent development of asthma^5, 17–19^. Thus, knowledge of the sensitization patterns to various allergens may provide a means to establish AR management strategy for allergists and rhinologists.

Skin prick tests (SPT) are the standard tool for routinely identifying sensitizing allergens for AR. For example, SPT has shown house dust mite (HDM) to be the main allergen for AR in Korea^14^, whereas pollens were more prevalent in Europe^20^. Despite the importance of SPT as a routinely employed test in daily clinical practice, this is of limited value when sensitizing allergens are missing from the screening panel which should account for regional differences.

Li and colleagues^5^ assessed the prevalence of sensitizations by SPT in patients with asthma and/or rhinitis in 17 Chinese cities from 2006 to 2007, and demonstrated significant differences in patterns of sensitizations from different geographical areas, age groups as well as asthma and/or rhinitis. However, this study did not define the minimal number and types of SPT allergens required to identify a patient as sensitized. Therefore, the present study was designed to firstly investigate the prevalence and novel patterns of sensitization in AR patients from 28 provinces within China and secondly analyze the clinical relevance of SPT results. Last but not least, based on these findings, we aimed to develop optimal SPT panels as a cost-effective tool for screening AR in the different regions.

## Results

### Demographic characteristics

Table 1 shows the demographic characteristics of the study population. A total of 7148 subjects with self-reported AR were enrolled in the study over a period of 31 months. The subjects were aged 6–75 years old, and 3803 (53.2%) were male and 3345 (46.8%) were female. The majority of subjects were from central China, with approximately equal numbers, coming from the other three regions.

**Table 1:** Demographic characteristics of study population.

Patients	North-East	North-West	Central China	South China	China
Frequency (n)	290	346	6236	276	7148
Percentage (%)	4.1	4.8	87.2	3.9	100.0
Gender					
*Male* (*n*, *%*)	166 (57.2)	198 (57.2)	3283 (52.6)	156 (56.5)	3803 (53.2)
*Female* (*n*, *%*)	124 (42.8)	148 (42.8)	2953 (47.4)	120 (43.5)	3345 (46.8)
Age groups (years, %)					
≤*14*	28 (9.7)	28 (8.1)	361 (5.8)	35 (12.7)	452 (6.3)
*15–29*	82 (28.3)	106 (30.6)	2188 (35.1)	103 (37.3)	2479 (34.7)
*30–44*	113 (39.0)	146 (42.2)	2629 (42.2)	101 (36.6)	2989 (41.8)
*45–*60	60 (20.7)	61 (17.6)	951 (15.3)	33 (12.0)	1105 (15.5)
>*60*	7 (2.4)	5 (1.4)	107 (1.7)	4 (1.4)	123 (1.7)

### Differences and standardization of sensitization rates (SRs)

**Figure 1:**
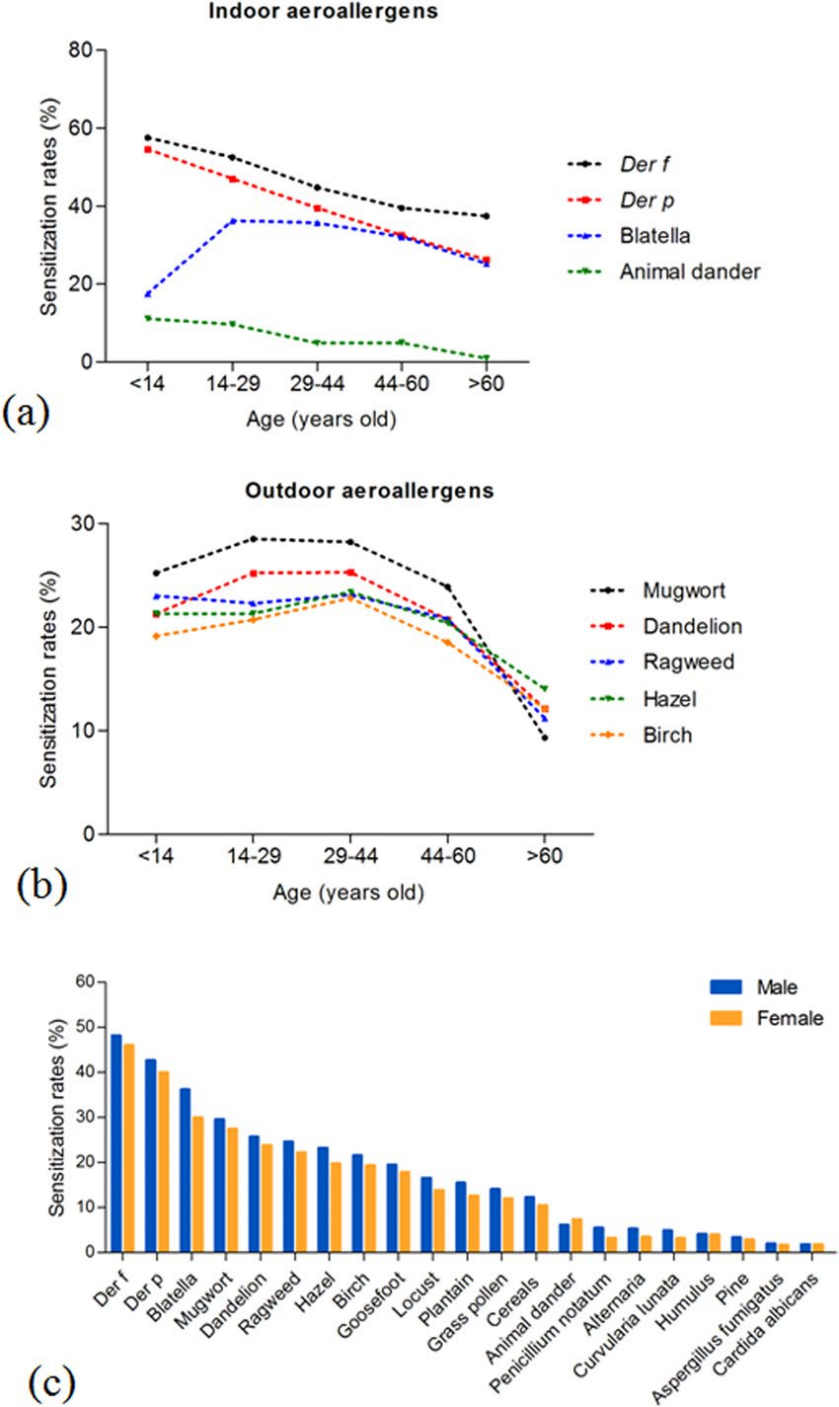
Effect of age and gender on sensitization rates (SRs) for different allergens. SRs for common indoor (**a**) and outdoor (**b**) aeroallergens changed with age in central China, and apart from animal dander (**c**), the SRs for majority of allergens were higher in males than in females.

Overall, 6340/7148 patients (88.7%) had at least one positive skin prick reaction. The proportion of sensitized patients among the self-reported patients in the different regions ranged from 85.9% (north-east China) to 90.6% (south China). *Der f* and *Der p* were found to be the most prevalent aeroallergens in China, and demonstrated standardized sensitization rates of 47.2% and 41.4%, for *Der f* and *Der p*, respectively (Table 2). Sensitizations to common allergens demonstrated unique patterns with age in patients from central China (Fig. 1a,b) and varied widely between the geographic areas (Table 2, Supplementary Fig. S1).

**Table 2:** Region-specific sensitization rates to inhalant allergens.

Region	Sensitization rates [%]							
	*Der f* SSR (95% CI)	*Der p* SSR (95% CI)	Animal dander SSR (95% CI)	*Blatella* SSR (95% CI)	Mugwort SSR (95% CI)	Dandelion SSR (95% CI)	Ragweed SSR (95% CI)	Hazel SSR (95% CI)
Mainland China	47.2 (46.0–48.3)	41.4 (40.3–42.6)	6.8 (6.2–7.4)	33.3 (32.2–34.4)	28.6 (27.6–29.7)	24.9 (23.9–25.9)	23.5 (22.5–24.5)	21.7 (20.7–22.6)
North-East	43.1 (37.4–48.8)	38.8 (33.2–44.4)	7.9 (4.8–11.0)	23.5 (18.6–28.4)	33.6 (28.2–39.0)	28.3 (23.1–33.5)	29.0 (23.7–34.2)	19.9 (15.3–24.5)
North-West	24.2 (19.7–28.7)	18.3 (14.2–22.4)	5.2 (2.9–7.5)	21.0 (16.7–25.3)	58.2 (53.0–63.4)	45.3 (40.1–50.6)	47.1 (41.8–52.3)	28.7 (23.9–33.5)
Central China	47.3 (46.1–48.6)	41.7 (40.5–43.0)	6.8 (6.2–7.4)	34.0 (32.8–35.2)	27.1 (26.0–28.2)	24.1 (23.0–25.1)	22.2 (21.2–23.3)	22.0 (20.9–23.0)
South China	69.2 (63.8–74.7)	61.4 (55.7–67.2)	7.6 (4.5–10.8)	39.4 (33.6–45.1)	14.9 (10.7–19.1)	11.7 (7.9–15.5)	14.3 (10.2–18.5)	7.7 (4.6–10.9)
**Region**	**Sensitization rates** [**%**]							
	**Birch SSR** (**95% CI**)	**Goosefoot SSR** (**95% CI**)	**Locust SSR** (**95% CI**)	**Plantain SSR** (**95% CI**)	**Grass pollen SSR** (**95% CI**)	**Cereals SSR** (**95% CI**)	**Pine SSR** (**95% CI**)	**Humulus SSR** (**95% CI**)
Mainland China	20.6 (19.6–21.5)	18.8 (17.9–19.7)	15.3 (14.5–16.2)	14.2 (13.4–15.0)	13.1 (12.3–13.9)	11.5 (10.7–12.2)	3.2 (2.8–3.6)	4.1 (3.6–4.5)
North-East	18.9 (14.4–23.4)	14.9 (10.8–19.0)	13.8 (9.8–17.7)	12.2 (8.4–15.9)	9.4 (6.1–12.8)	7.9 (4.8–11.0)	3.2 (1.1–5.2)	4.8 (2.3–7.2)
North-West	24.2 (19.7–28.8)	38.3 (33.1–43.4)	29.3 (24.5–34.1)	27.4 (22.7–32.1)	26.7 (22.1–31.4)	25.7 (21.1–30.3)	4.1 (2.0–6.1)	8.2 (5.3–11.1)
Central China	21.0 (20.0–22.0)	18.6 (17.6–19.5)	15.0 (14.1–15.9)	14.0 (13.1–14.9)	12.8 (12.0–13.7)	11.0 (10.2–11.7)	3.1 (2.7–3.6)	4.0 (3.5–4.4)
South China	7.6 (4.5–10.7)	5.4 (2.7–8.1)	7.8 (4.7–11.0)	5.5 (2.8–8.1)	6.4 (3.5–9.3)	7.3 (4.2–10.4)	2.1 (0.4–3.8)	1.1 (−0.1–2.3)
**Region**	**Sensitization rates** [**%**]							
	***Aspergillus fumigatus*** **SSR** (**95% CI**)	***Alternaria spec***. **SSR** (**95% CI**)	***Curvularia lunata*** **SSR** (**95% CI**)	***Penicillium notatum*** **SSR** (**95% CI**)				
Mainland China	1.9 (1.6–2.2)	4.4 (4.0–4.9)	4.1 (3.6–4.6)	4.5 (4.0–4.9)				
North-East	1.3 (0.0–2.6)	3.8 (1.6–6.0)	4.4 (2.0–6.7)	3.1 (1.1–5.1)				
North-West	1.0 (0.0–2.1)	3.7 (1.7–5.7)	4.3 (2.2–6.4)	2.8 (1.1–4.6)				
Central China	1.9 (1.6–2.2)	4.6 (4.1–5.1)	4.1 (3.6–4.6)	4.5 (4.0–5.0)				
South China	2.4 (0.6–4.2)	3.5 (1.3–5.7)	3.4 (1.3–5.5)	5.7 (2.9–8.4)				

SSR, standardized sensitization rate adjusted for gender and age.

In central China, sensitization to prevalent indoor allergens diminished with age (Fig. 1a). HDMs showed the highest prevalence in all age groups. In addition, *Der f* was more prevalent than *Der p*. Sensitization of *Blatella* peaked in the age group of 14–29 years and then decreased with age, showing a higher incidence than animal dander. The prevalence of sensitization to common outdoor allergens revealed no obvious fluctuations from age group 0–14 years to age group 29–44 years, and then decreased progressively (Fig. 1b). The SR of mugwort was higher than other outdoor allergens throughout the age groups in central China; however, the overall percentages of five common outdoor allergens (mugwort, dandelion, ragweed, hazel, birch) gradually decreased to a lower percentage at over 60 years of age. Sensitization rate of any type of mold was less than 5%, which was markedly lower than for the majority of indoor and outdoor allergens (Table 2). The sensitization to *Alterneria* fell sharply from the youngest age group to the 29–44 year age group, and sensitization to *Culvularia lunata* mirrored this trend. When the difference in gender was considered, the SRs of most aeroallergens were higher among males than among females in the general population; with the exception of animal dander, which showed lower prevalence of sensitization in males (Fig. 1c).

In view of the age and gender-associated differences, the region-specific SRs were standardized for age and gender to investigate local sensitization patterns. In this regard, the highest SRs for HDMs were observed in south China (69.2% for *Der f* and 61.4% for *Der p*, respectively) (Table 2); where the climate was characterized by warm temperatures, high humidity and hot summers. However, in north-west China with the cold arid and desert/steppe climate, the three most prevalent aeroallergens were mugwort, ragweed and dandelion pollen (58.2%, 47.1% and 45.3%, respectively) (Table 2, Supplementary Fig. S1). These results supported the notion that local allergens had an important impact on the establishment of region-specific sensitization pattern.

### Sensitization and AR persistence/severity

Sensitization to HDMs was not significantly correlated with the persistence or severity of AR; however, SRs of common outdoor aeroallergens were significantly higher in patients with persistent AR, compared to patients with intermittent AR (*P* < 0.01 for ragweed, birch, goosefoot and grass pollen, and *P* < 0.05 for cereals) in central China (Supplementary Table S1). Similarly, significantly higher SRs of mugwort and dandelion were observed in patients with moderate/severe AR compared to patients with mild AR (*P* = 0.002 for both allergens) (Supplementary Table S1).

### Allergen sensitization and comorbidities

There were a total of 1081 patients (17.0%) with mono-sensitization, 1567 patients (24.7%) with 2 sensitizations, 1252 patients (19.7%) with 3 sensitizations, 631 patients (9.94%) with 4 sensitizations and 1819 patients (28.6%) with 5 or more sensitizations. The cumulative numbers of allergens were associated with AR comorbidities (Supplementary Fig. S2). In patients with AR alone the percentages of mono-sensitized (17.4%), double sensitized (25.1%), and triple sensitized (19.8%) patients were significantly higher than in patients with AR and asthma (11.3% (*P* < 0.01), 17.9% (*P* < 0.01), and 16.2% (*P* < 0.05) in mono-sensitized, double sensitized and triple sensitized patients, respectively) and in patients with AR and conjunctivitis (10.7% (*P* < 0.01), 17.4% (*P* < 0.01), and 17.4% (*P* < 0.05) in mono-sensitized, double sensitized and triple sensitized patients, respectively) (Supplementary Fig. S2). Conversely, a significantly lower percentage of 5 or more allergen-sensitizations was observed in patients with AR alone (27.8%) compared to patients with AR and asthma (37.7%; *P* < 0.001) and patients with AR and conjunctivitis (37.2%; *P* < 0.001) (Supplementary Fig. S2); indicating that poly-sensitization to more than 5 types of allergens might predispose the subjects to development of comorbid asthma and conjunctivitis in AR patients. Indeed, the distribution of the number of sensitizing aeroallergens in patients with AR and asthma was comparable to that in patients with AR and conjunctivitis.

Patients with AR and asthma had significantly higher positive sensitization rates to common outdoor allergens and animal dander (40.4%, 28.8%, 35.4%, 31.5% and 18.9% for mugwort, hazel, dandelion, ragweed, and animal dander, respectively) than patients without asthma (28.1% (*P* < 0.001), 21.3% (*P* = 0.002), 24.4% (*P* < 0.001), 23.2% (*P* = 0.001) and 6.3% (*P* < 0.001) for mugwort, hazel, dandelion, and ragweed, and animal dander, respectively) (Supplementary Table S2). Similarly, sensitization against the majority of inhalant allergens was significantly increased in patients with AR and conjunctivitis, compared to patients without conjunctivitis (Supplementary Table S2), whereas sensitization to *Der f*, *Der p* and *Blatella s*howed no difference between the two groups. Sensitization to common outdoor allergens is prone to be detected by SPT in patients with AR and comorbidities, compared to AR without comorbidities.

### Optimization of skin prick test panel

Overall, eight allergens (*Der f*, mugwort, *Blatella*, hazel, goosefoot, *Penicillium notatum*, animal dander and *Der p*) enabled the identification of >96% of sensitized subjects for AR in central China (Table 3). Overall, 2950 patients (47.3% of 6236 self-reported AR patients in central China) positive to *Der f* could be detected by the first allergen in the panel. In the second step, 1028 (16.48%) patients sensitized to mugwort were identified in patients negative to *Der f*, and then 3978 (63.8%) patients with sensitization to either *Der f* or mugwort were confirmed. By this process, after *Der p*, the eighth allergen in the panel, to which 5313 (85.2%) patients were sensitized, this accounted for more than 96% (5313/5530) of all sensitized subjects in the group from central China (Table 3). The nine allergens (grass pollen, ragweed, locust, *Curvularia lunata*, plantain, *Aspergillus fumigatus*, humulus, cereals, and pine) induced <0.5% increase in sensitization prevalence over the eight allergens tested, and were not included as part of the optimal allergens panel. The influence of age and gender was taken into consideration when designing the minimal antigen battery in central China (Table 4). *Der f*, mugwort and *Blatella* were included as the commonest screening antigens in most batteries of different age and gender groups.

**Table 3:** Prevalence of sensitization; allergens ordered from the most prevalent to the allergen with the least increase in identifying additional sensitized subjects in central China.

Allergen	Prevalence	Increase in identifying sensitized patients by adding allergen in skin prick test	
	n (%)	n	%
*Der f*	2950 (47.3)		
Mugwort	3978 (63.8)	1028	16.48
*Blatella*	4593 (73.7)	615	9.86
Hazel	5959 (79.5)	366	5.87
Goosefoot	5080 (81.5)	121	1.94
*Penicillium notatum*	5166 (82.8)	86	1.38
Animal dander	5241 (84.0)	75	1.20
*Der p*	5313 (85.2)	72	1.15
Birch	5372 (86.2)	59	9.46
*Alternaria*	5412 (86.8)	40	0.64
Dandelion	5444 (87.3)	32	0.51
Grass pollen	5468 (87.7)	24	0.38
Ragweed	5485 (88.0)	17	0.27
Locust	5499 (88.2)	14	0.23
*Curvularia lunata*	5510 (88.4)	11	0.18
Plantain	5516 (88. 5)	6	0.09
*Aspergillus fumigatus*	5522 (88.6)	6	0.09
Humulus	5526 (88.6)	4	0.06
Cereals	5529 (88.7)	3	0.05
Pine	5530 (88.7)	1	0.01

Grey shaded cells: More than 96% of sensitized subjects identified.

**Table 4:** Minimal antigen panels according to age and gender in central China; allergens order from the most prevalent to the allergen with the less increase in identifying additional sensitized subjects.

Patients (gender, age)	Allergens								
	1	2	3	4	5	6	7	8	9
Male, ≤14yrs	*Der f*	Mugwort	*Alternaria*	*Blatella*	Hazel				
Female, ≤14yrs	*Der f*	Hazel	*Blatella*	Goosefoot	*Alternaria*	Mugwort	Animal dander	*Der p*	
Male, 15–29 yrs	*Der f*	Mugwort	*Blatella*	Hazel	*Alternaria*	Animal dander			
Female, 15–29 yrs	*Der f*	Mugwort	*Blatella*	Hazel	Animal dander	Goosefoot	*Der p*		
Male, 30–44 yrs	*Der f*	Mugwort	*Blatella*	Birch	*Penicillium notatum*	Goosefoot	Hazel		
Female, 30–44 yrs	*Der f*	Mugwort	*Blatella*	Hazel	Goosefoot	Birch	*Der p*	*Penicillium notatum*	
Male, 45–60 yrs	*Der f*	Mugwort	*Blatella*	Hazel	*Penicillium notatum*	Goosefoot	Animal dander		
Female, 45–60 yrs	*Der f*	Mugwort	*Blatella*	Hazel	Goosefoot	Ragweed	Gross pollen	*Der p*	*Penicillium notatum*
Male, >60 yrs	*Der f*	Ragweed	*Blatella*	*Penicillium notatum*	Hazel	Mugwort	Dandelion	Pine	
Female, >60 yrs	*Der f*	Dandelion	*Blatella*	Locust	*Penicillium notatum*	Animal dander			

Cells in gray: More than 96% of sensitized subjects are identified.

Using the same allergen panel as for central China, >96% of the sensitized subjects could be screened for AR in the other regions. A minimum of 8 allergens were required for north-east China, a minimum of 5 allergens for north-west China, and a minimum of 7 allergens for south China (Table 5); indicating that the optimal panel for central China could be applied across China. However, differences were observed when performing the conditional approach analysis at a region-specific level, which demonstrated that only 5 allergens were sufficient for screening AR in south China (*Der f*, *Blatella*, dandelion, ragweed and birch) and north-west China (mugwort, *Der f*, goosefoot, *Blatella* and hazel), whereas 6 aeroallergens, including *Der f*, mugwort, *Blatella*, hazel, goosefoot and dandelion, were sufficient for screening AR in north-east China (Table 6).

**Table 5:** Prevalence of sensitization in three other regions with the same order of allergens as used in central China.

Region	n	Allergens												
		1	2	3	4	5	6	7	8	9	10	11	12	13
North-East	290	139 (47.9%)	56 (67.2%)	19 (73.8%)	12 (77.9%)	7 (80.3%)	3 (81.4%)	3 (82.4%)	2 (83.1%)	1 (83.4%)	0 (85.5%)	6 (85.5%)	0 (85.5%)	1 (85.9%)
North-West	346	85 (24.6%)	158 (70.2%)	21 (76.3%)	9 (78.9%)	23 (85.5%)	3 (86.4%)	2 (87.0%)	1 (87.3%)	0 (87.3%)	1 (87.6%)	2 (88.2%)	0 (88.2%)	0 (88.2%)
South China	276	197 (71.4%)	15 (76.8%)	20 (84.1%)	4 (85.5%)	0 (85.5%)	1 (85.9%)	2 (86.6%)	1 (87.0%)	1 (87.3%)	1 (87.7%)	3 (88.8%)	1 (89.1%)	2 (89.9%)
**Region**	**Allergens**													
	**14**	**15**	**16**	**17**	**18**	**19**	**20**							
North-East	*	*	*	*	*	*	*							
North-West	3 (88.7%)	1 (89.3%)	1 (89.6%)	0 (89.6%)	1 (89.9%)	*	*							
South China	1 (90.2%)	0 (90.2%)	0 (90.2%)	1 (90.6%)	*	*	*							

Allergens tested were as follows: 1, *Der f*; 2, Mugwort; 3, Blatella; 4, Hazel; 5, Goosefoot; 6, *Penicillium notatum*; 7, Animal dander; 8, *Der p*; 9, Birch; 10, *Alternaria*; 11, Dandelion; 12, Grass pollen; 13, Ragweed; 14, Locust; 15,*Curvularialunata*; 16, Plantain; 17, *Aspergillus fumigatus*; 18, Humulus; 19, Cereals; 20, Pine.

Results are expressed as frequencies and percentages.

Grey shaded cells: More than 96% of sensitized subjects are identified.

*Additional allergen did not change the prevalence.

**Table 6:** Optimal screening panels according to region; allergens order from the most prevalent to the allergen with the least increase in identifying additional sensitized subjects under condition for each region.

Region	Allergens							
	1	2	3	4	5	6	7	8
North-East	*Der f*	Mugwort	*Blatella*	Hazel	Goosefoot	Dandelion		
North-West	Mugwort	*Der f*	Goosefoot	*Blatella*	Hazel			
Central China	*Der f*	Mugwort	*Blatella*	Hazel	Goosefoot	*Penicillium notatum*	Animal dander	*Der p*
South China	*Der f*	*Blatella*	Dandelion	Ragweed	Birch			

Grey shaded cells: More than 96% of sensitized subjects are identified.

## Discussion

The study was conducted over 31 months to omit seasonal biases and allow a better surveillance of inhalant allergen sensitization in a large, geographically representative population of AR patients in areas with different climatic conditions within China. Our results demonstrated that the sensitization profile in AR patients varied widely between regions, differed according to age and gender, and correlated with concomitant allergic diseases. More importantly, this study demonstrated that a SPT panel comprising only eight specific allergens; including *Der f*, mugwort, *Blatella*, hazel, goosefoot, *Penicillium notatum*, animal dander and *Der p*; could be used as a cost-effective tool for sensitization screening of AR in China.

In accordance with previous studies in Chinese^5, 21^ and Korean^14, 22^ subjects, our study also demonstrated that HDM allergens, *Der f* and *Der p*, were the most prevalent sensitizing aeroallergens, and particularly abundant in environment with ambient humidity and higher temperature. In northern and western China, HDM allergen levels have generally been shown to be approximately 50 times lower than those in southern China^23^. Correspondingly, in the present study, the maximum sensitization rates of 69.2% and 61.4% for *Der f* and *Der p*, respectively, were found in south China, with its humid and hot climate, while the lowest prevalence of sensitization rates of 24.2% and 18.3% for *Der f* and *Der p*, respectively, were found in north-west China, with its arid, desert/steppe, cold arid climate. Indeed, a similar trend was also noted for sensitization rates to moulds, including *Aspergillus fumigatus* and *Penicillium notatum*, which was consistent with increasing temperature and humidity from the north to south. Patients in north-west China with the desert/steppe, cold arid climate had significantly higher positive rates to pollens than in the other three regions; with sensitization to mugwort being the most prominent (58.2%). This finding is also in accordance with those of studies conducted in dry areas, which have reported sensitization to pollens from weeds, grasses and trees to be the most prevalent in AR patients^24, 25^. Collectively, these findings support the notion that geographical and climatic differences result in variable exposure to sensitizing inhalant allergens and subsequently to differences in sensitization rates to these allergens.

Our study has suggested that besides the environment, age and gender also appear to influence the sensitization to inhalant allergens; with increment of age and female gender being less sensitive to the majority of the common aeroallergens investigated in the present study. Highest prevalence of sensitization to most indoor allergens, including *Der f*, *Der p*, animal dander and common moulds, was seen in the youngest age group and the sensitization rates declined progressively with increment of age. Furthermore, pollen sensitization was noted predominantly in young and middle age patients. This finding is also in concordance with findings of several studies^14, 17, 25–27^, which showed that younger patients were more sensitive than older patients. Similarly, other studies have also demonstrated a predominance of sensitization to inhaled allergens in males^12, 13, 25^. Indeed, the mean total IgE serum was also shown to be significantly greater in men than women^25^. Whether the differences between young and aged and between male and female was due to the tolerance of immune system or reflect different allergen exposure level needs further exploration.

In addition to the type of allergen, the presence of poly-sensitization also represents a risk factor for the development of allergic disease in sensitized patients. Several studies have demonstrated that an increasing number of sensitizations strongly predisposed individuals to the presence of AR symptoms^13, 17, 19^. Moreover, patients with AR and multiple comorbidities have been shown to have markedly higher prevalence of sensitizations to the tested allergens and concomitant reactivity to multiple allergens than patients with AR alone^5, 18^. In line with these studies^5, 18^, outdoor allergens were also more likely to affect patients with rhinitis and conjunctivitis, while indoor allergens, especially mites, affected patients with asthma^10, 19, 23^.

Multi-allergen sensitization may be caused by parallel sensitization or cross- reactivity among closely related allergens. It is likely that the immune system cannot distinguish *Der p* or *Der f* allergens due to their high degree of homology between these allergens^5^. Among the 3631 HDM-sensitized patients enrolled in the present study, 2663 patients were concomitantly allergic to both *Der f* and *Der p* (73.7%). Similarly, the GA^2^LEN study has shown that reactions to the two mites were concordant in 80% of patients^20^. *Der f*, which is slightly more prevalent than *Der p*, was first used as a screening allergen in our approach to compose the minimal SPT panel. However, the incidence of *Der p* sensitization was not high in patients who showed a negative response to *Der f* because of co-sensitization, and thus *Der p* was not included as a screening allergen in the optimal panels for north-east, north-west, or south China, based on its ability to increase sensitization prevalence.

Due to the potential cross-reactivity between aeroallergens, great effort was made to define the minimal SPT battery to detect maximal sensitization. Employing a step-by-step conditional approach, our study demonstrated that a minimum of eight region-related allergens was adequate for identifying 96% of sensitized patients from four major regions in China determined according to the Köppen-Geiger climate map^28^. With respect to region-specific sensitization patterns, mugwort was incorporated first into north-west China screening panel as the most prevalent allergen in the local area. When the predominant allergen *Der f* accounted for 71.4% of sensitized patients in south China, the number of allergen extracts in the test panel could be reduced to five to identify 96% of sensitized patients. Conversely, a decline in the prevalence of sensitization to the predominant allergen led to an increase in the number of minimum allergens in the SPT panel.

The step-by-step conditional approach was first developed by Bousquet and colleagues^20^ to select optimal allergens from a standardized panel of 18 allergens, which would allow identification of at least 95% of all sensitized subjects by skin prick testing in a large Pan-European multicenter (17 centers in 14 countries) study (GA2LEN skin test study III). These authors demonstrated that eight of these allergens (grass pollen, *Der p*, birch pollen, cat dander, *Artemisia*, olive pollen, *Blatella* and *Alternaria*) allowed to identify more than 95% of sensitized subjects, although up to 13 allergens were required to identify all the sensitized subjects. However, differences were observed between countries, with two allergens being sufficient for Switzerland (grass pollen and cat dander), as compared to nine for France (grass pollen, *Der p*, olive pollen, cat dander, *Blatella*, cypress, dog dander, alder and [*Artemisia* or *Alternaria*]).

The findings from the present study, which is the first study in China to identify optimal allergen panels for sensitization screening by SPT, are in accordance with the findings of the GA2LEN skin test study^20^. In particular, our study also demonstrated that a minimum of eight allergens (*Der f*, mugwort, *Blatella*, hazel, goosefoot, *Penicillium notatum*, animal dander and *Der p*) were required to identify more than 96% of sensitized subjects, and the number and the type of allergens were highly correlated to the region, thus demonstrating the importance of selecting geographically appropriate allergens for optimizing the SPT panels. Moreover, in Europe while pollens exerted the greatest sensitization impact^20^, in China *Der f* was the main offending allergen. However, unlike the GA2LEN skin test study^20^, the present study has additionally assessed the impact of gender and age, in view of potential to modify allergen sensitization patterns^12–16^. In this regard, our study is in accordance with the study of Lee and colleagues^14^, which employed the step-by-step conditional approach to identify optimal offending allergen panels for sensitization screening of AR by SPT in Korean subjects in different age groups^14^. These authors demonstrated that testing with five to seven allergens (*Der f*, *Tetranychus urticae*, oak, mugwort, cockroach, *Der p* and cat) adequately identified over 93–95% of the sensitized patients. Similar to the present study, *Der f* was also found to have the most profound sensitization in the Korean subjects, whereas *Der f* and *Der p* were the most prominent allergens. Similarly, the prevalence of sensitization to common allergens increased with age, from group 0–6 years to group 13–19 years, and then decreased progressively. The present study has also demonstrated a similar age-related effect, although sensitization to especially outdoor allergens appears to progressively decrease after 29–44 years. The study by Lee and colleagues^14^, however, did not investigate the effect of gender. Collectively, these findings support the view that relevant allergens in the panels should be optimized with respect to a specific age and gender brackets for accuracy.

In china, imbalance in provision of medical service has resulted in accumulation of patients from the countryside to central cities, such as Beijing. In this regard, nearly 30% of AR patients from faraway areas seek medical help in our allergy-rhinology center, which is qualified with international certification. This single center study is thus limited by the possible selection bias of recruited patients, particularly as only the patients currently living in their hometown were included to represent local allergy characteristics. Despite this limitation, the sensitization patterns noted in different geographic areas of China in our study were found to be similar to those reported by Li and colleagues, employing a multiple-center study design^5^. This suggests that the recruited patients in the present study can be taken as being representative of the general populations in different areas.

In conclusion, this patient-based study revealed that *Der f* and mugwort were the most prevalent indoor and outdoor sensitizing allergens, respectively, for AR throughout China. Significant differences in patterns of sensitizations were confirmed between different geographic areas, age groups and genders. With regard to specific regions, a limited allergen panel of five to eight allergens allowed the identification of 96% of sensitized subjects. These findings suggest that optimal SPT panels have the potential for accurately screening large numbers of sensitized patients for AR throughout China with great cost-savings, due to the very limited numbers of allergens required, as well as the ease and simplicity with which the SPT can be performed. Moreover, these panels have the potential to provide clinicians with important clinical pointers for the presence of allergic comorbidities (particularly asthma and conjunctivitis), disease severity and disease persistence, thus leading to better overall management of AR patients. Moreover, the findings from the current study support previous findings that gender and age are both likely to influence SPTs, and thus limited selected allergen panels should be developed and employed in consideration of both gender and age category for screening of AR.

## Patients and Methods

### Study design and settings

This study was conducted from January 2011 to July 2013 in a single center. The study participants suspected to be suffering from inhalant allergy, based on presence of symptoms of sneezing, nasal itching, rhinorrhea and nasal blockage, were recruited consecutively in allergy-rhinology clinic of Beijing TongRen Hospital and assessed for sensitization to relevant allergens by SPT. The patients came from 28 provinces in China, where they had lived since birth, and visited our center for better medical help. The recruited subjects were classified into four regional groups according to the Köppen-Geiger climate map^28^. North-east China was defined as Dwa (snow, winter dry, hot summer), north-west China as BWk/BSk (arid, desert/steppe, cold arid), central China as Cwa (warm temperature, winter dry, hot summer), and south China as Cfa (warm temperature, fully humid, hot summer).

Diagnosis of AR was confirmed based on the presence of symptoms as noted above and clinically relevant SPT results. Questionnaires were completed by each participant at recruitment to record demographic data, nasal symptom severity, and physician-diagnosed comorbidities (allergic conjunctivitis and asthma). Phenotype of AR was classified according to the ARIA classification^1^.

The study was conducted in full accordance with Declaration of Helsinki and approved by the Ethics Review Board of Beijing TongRen Hospital. Written informed consent was obtained from each participant prior to entry in the study.

### Allergen extracts and reagents

A standard skin prick testing core panel comprising the 20 most frequent inhalant allergens within mainland China was agreed upon, taking into consideration sensitization rates, Chinese ecology, climate and cross-reactivity. The selected test inhalant allergens (Allergopharma GmbH & Co. KG, Reinbek, Germany) were animal dander (cat, dog), hazel, birch, grass pollen, cereals, mugwort, dandelion, ragweed, goosefoot, *Humulus*, locust, *Blatella*, pine, plantain, *Curvularia lunata*, *Penicilliumnotatum*, *Alternaria*, *Aspergillus fumigatus*, *Dermatophagoides farinae* (*Der f*) and *Dermatophagoides pteronyssinus* (*Der p*). Histamine (10 mg/mL) and saline were used as positive and negative controls, respectively.

### SPT

SPT was performed according to a standardized protocol^4^. All the participants were instructed to abstain from taking any antihistamines for at least 72 hours before examination. The development of a wheal in response to the allergen was assessed 15 min after application of the allergen and the wheal size was calculated as the mean of the longest diameter and the length of the perpendicular line through its middle. A wheal reaction of greater than 3mm to histamine was considered as a positive control, and employed to calculate the skin index (SI)^5^; as a ratio of allergen wheal size to histamine wheal size. SI was expressed as 2+ when the wheal diameter was half that of the positive control, 3+ when the mean wheal diameter was identical to the positive control, and 4+ when the mean wheal diameter was double that of the positive control. Atopy was defined as positive when the SI was ≥2+ for at least one allergen.

### Study size

We have previously reported that the positive SPT rate among subjects with self-reported AR was 32.5% in Baoding, in central China; with Cwa climate^29^. In the present study, we estimated that to screen >96% sensitized patients, with a 4% margin of error and a 2-tailed α value of 0.05, the size of the cohort in central China needed to be 4987. Considering a dropout of 25% participants because of incomplete SPT data in clinical practice, we recruited 6649 participants. However, 413 of these patients were excluded due to availability of insufficient SPT data on the total 20 aeroallergens assessed in the present study. Thus, 6236 patients from central China were tested to initially obtain the optimal aeroallergen panel for this area, and the feasibility of this panel was further tested in patients from the other three areas.

### Statistical analysis

Statistical analysis was performed using the SPSS version 22.0 software package (IBM Corp., Armonk, NY, USA). The regional study populations were standardized by adjusting for age and gender and the entire study population was used as the reference population. The weight of patients in each gender and age group was calculated in all the patients of the study population (Supplementary Table S3). Sensitization to specific allergens in each region was calculated as the percentage of total sensitized patients and expressed as standardized sensitization rates (SSRs) and 95% confidence intervals (CIs). Chi-square test was used to compare the differences in SSRs between geographical areas, genders, age groups, and AR phenotypes with/without comorbidities. Statistical significance was set at a *P* value ≤ 0.05.

A step-by-step conditional approach, which allowed to determine the selection of allergens from the one that gave the highest increase in prevalence of sensitization to the one that gave the lowest, was adopted as described by Bousquet and colleagues^20^. In this regard, the allergen panel was started with the most prevalent allergen and the next allergen was identified by a process of elimination, in which the subjects sensitized to the first allergen was excluded and the second allergen in the panel was determined as the most prevalent sensitizing allergen in the remaining group of patients. Increase in identifying sensitized patients by adding the second allergen in SPT panel did not include the subjects co-sensitized to both the first and the second allergens, as they were previously detected by the first allergen in the optimal panel. Similarly, the third allergen in the panel was determined as the one that gave the highest probability of sensitization knowing the subjects were not sensitized to either the first or the second allergen. The procedure was repeated until none of the remaining allergens induced an increase in prevalence. The combination of allergens that identified at least 96% of all sensitized subjects was defined as the optimal panel.

## Electronic supplementary material

Supplementary Tables and Figures
