# Supplementary material for: Sensitization patterns and minimum screening panels for aeroallergens in self-reported allergic rhinitis in China
Source: Sci Rep. 2017 Aug 24;7:9286. doi: 10.1038/s41598-017-10111-9 (PMC5570894; doi:10.1038/s41598-017-10111-9)
Supplement: Supplementary file 1 — Supplementary Tables and Figures [file 41598_2017_10111_MOESM1_ESM.pdf]

Title: Sensitization patterns and minimum screening panels for aeroallergens in self-reported allergic rhinitis in China

Authors: Hongfei Lou[1], Siyuan Ma[1], Yan Zhao[2], Feifei Cao[3], Fei He[2], Zhongyan Liu[2], Jean Bousquet[4], Chengshuo Wang[1]*, Luo Zhang[1,2,3]*, Claus Bachert[5]

*: These authors contributed equally to the study.

Table S1. Sensitization rates of allergens in AR patients of different phenotypes.

Allergen	Sensitization rates (%)			
	Moderate/severe, persistent AR (N=3460)	Moderate/severe, intermittent AR (N=2245)	Mild persistent AR (N=604)	Mild intermittent AR (N=839)
Der f	46.2	48.5	50.0	49.9
Der p	40.3	43.5	40.3	42.8
Mugwort	30.8	27.9	24.7	24.0
Birch	22.5	19.4	21.7	17.0
Grass pollen	15.1	11.1	13.7	11.4
Dandelion	26.8	25.3	21.0	19.5
Ragweed	25.7	22.5	24.0	19.0
Goosefoot	20.3	18.1	19.7	15.3
Cereals	13.0	9.9	12.3	10.0

Table S2. Sensitization rates of allergens in AR patients with or without comorbid asthma or conjunctivitis

Allergen	Sensitization rates (%)		*P*	Sensitization rates (%)		*P*
	AR with asthma (N=302)	AR without asthma (N=6846)		AR with conjunctivitis (N=242)	AR without conjunctivitis (N=6906)	
Der f	53.0	46.9	0.038	49.2	47.1	0.523
Der p	47.4	41.2	0.033	42.6	41.4	0.715
Animal dander	18.9	6.3	<0.001*	10.3	6.7	0.026
Blatella	30.1	33.5	0.227	28.9	33.5	0.139
Mugwort	40.4	28.1	<0.001*	37.2	28.3	0.003*
Hazel	28.8	21.3	0.002*	33.5	21.2	<0.001*
Birch	24.2	20.4	0.115	36.4	20.0	<0.001*
Grass pollen	18.2	12.9	0.007	15.7	13.0	0.021
Cereals	16.6	11.2	0.005	16.1	11.3	0.227
Dandelion	35.4	24.4	<0.001*	35.1	24.5	<0.001*
Ragweed	31.5	23.2	0.001*	37.2	23.0	<0.001*
Goosefoot	20.9	18.7	0.343	31.8	18.3	<0.001*
Humulus	7.0	4.0	0.008	5.4	4.0	0.304
Locust	21.9	15.0	0.001*	23.1	15.0	<0.001*
Plantain	19.2	14.0	0.011	21.5	13.9	<0.001*
Pine	6.0	3.1	0.005	5.4	3.1	0.047

*Include a Bonferroni's correction, significance at *P* <0.003.

Table S3. Standardization weight in the reference population.

Patients		N	Weight
Age	Gender		
≤14	Male	292	0.040850588
	Female	160	0.022383884
15-29	Male	1310	0.183268047
	Female	1169	0.16354225
30-44	Male	1614	0.225797426
	Female	1375	0.1923615
45-60	Male	502	0.070229435
	Female	603	0.084359261
>60	Male	85	0.011891438
	Female	38	0.005316172

SUPPLEMENTARY FIGURES

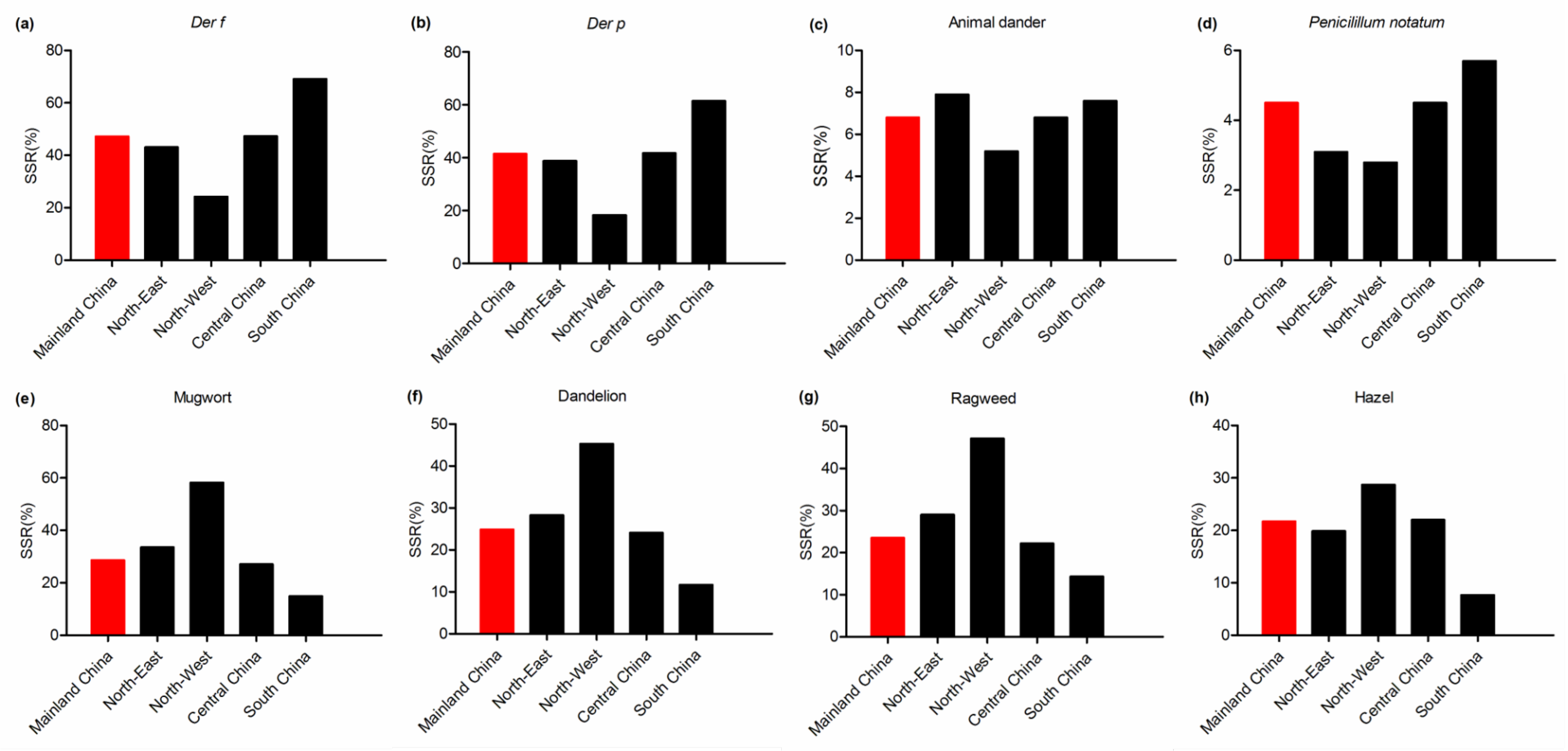

Figure S1. Standardized sensitization rates (SSRs) adjusted for age and gender in AR patients from geographic areas of China with different

climates. SSRs of indoor allergens (1a, *Der f*; 1b, *Der p*; 1c, animal dander; and 1d, *Penicilillum notatum*) were lowest in north-west China with desert/steppe and cold arid climate, and highest in humid and warm south China. Conversely, SSRs of outdoor allergens (1e, mugwort; 1f, dandelion; 1g, ragweed; and 1h, hazel) were highest in north-west China, and lowest in south China.

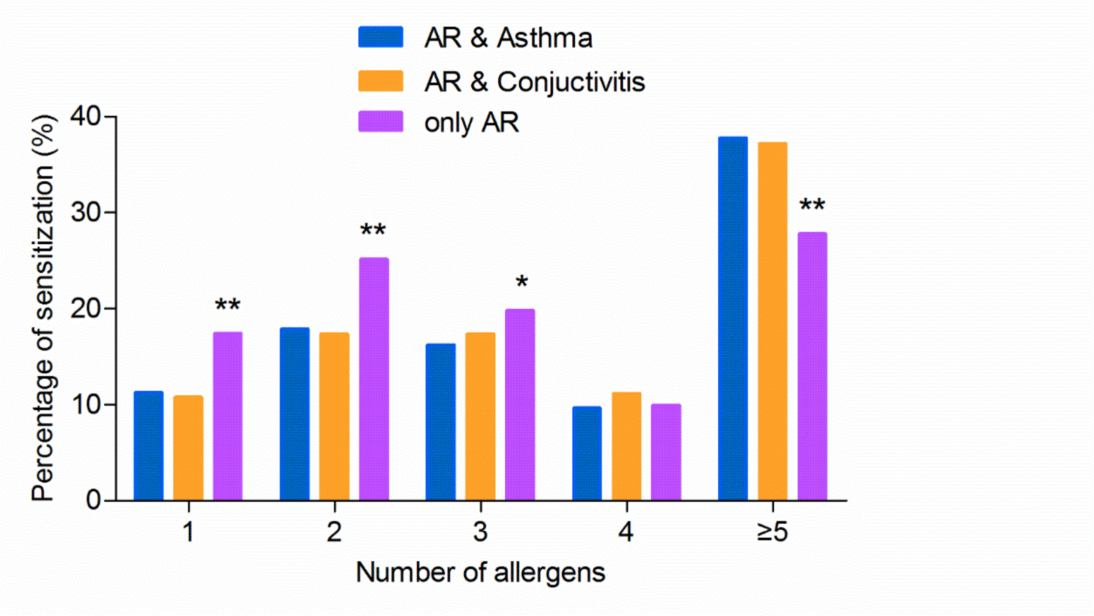

Figure S2. Distribution of the number of sensitizing allergens in patients with allergic rhinitis (AR), AR & asthma, and AR & allergic conjunctivitis. (** $P < 0.01$, * $P < 0.05$; both for AR vs AR and asthma/AR and conjunctivitis)
